# Limitations of insulin resistance assessment in polycystic ovary syndrome

**DOI:** 10.1530/EC-18-0021

**Published:** 2018-02-07

**Authors:** Krzysztof C Lewandowski, Justyna Płusajska, Wojciech Horzelski, Ewa Bieniek, Andrzej Lewiński

**Affiliations:** 1 Department of Endocrinology and Metabolic DiseasesMedical University of Lodz, Lodz, Poland; 2 Polish Mother’s Memorial Hospital–Research InstituteLodz, Poland; 3 Faculty of Mathematics and Computer ScienceUniversity of Lodz, Lodz, Poland

**Keywords:** polycystic ovary syndrome, insulin resistance, Belfiore index, Matsuda index, Stumvoll index, McAuley index, HOMA, QUICKI

## Abstract

**Background:**

Though insulin resistance (IR) is common in polycystic ovary syndrome (PCOS), there is no agreement as to what surrogate method of assessment of IR is most reliable.

**Subjects and methods:**

In 478 women with PCOS, we compared methods based on fasting insulin and either fasting glucose (HOMA-IR and QUICKI) or triglycerides (McAuley Index) with IR indices derived from glucose and insulin during OGTT (Belfiore, Matsuda and Stumvoll indices).

**Results:**

There was a strong correlation between IR indices derived from fasting values HOMA-IR/QUICKI, *r* = −0.999, HOMA-IR/McAuley index, *r* = −0.849 and between all OGTT-derived IR indices (e.g. *r* = −0.876, for IRI/Matsuda, *r* = −0.808, for IRI/Stumvoll, and *r* = 0.947, for Matsuda/Stumvoll index, *P* < 0.001 for all), contrasting with a significant (*P* < 0.001), but highly variable correlation between IR indices derived from fasting vs OGTT-derived variables, ranging from *r* = −0.881 (HOMA-IR/Matsuda), through *r* = 0.58, or *r* = −0.58 (IRI/HOMA-IR, IRI/QUICKI, respectively) to *r* = 0.41 (QUICKI/Stumvoll), and *r* = 0.386 for QUICKI/Matsuda indices. Detailed comparison between HOMA-IR and IRI revealed that concordance between HOMA and IRI was poor for HOMA-IR/IRI values above 75th and 90th percentile. For instance, only 53% (70/132) women with HOMA-IR >75th percentile had IRI value also above 75th percentile. There was a significant, but weak correlation of all IR indices with testosterone concentrations.

**Conclusions:**

Significant number of women with PCOS can be classified as being either insulin sensitive or insulin resistant depending on the method applied, as correlation between various IR indices is highly variable. Clinical application of surrogate indices for assessment of IR in PCOS must be therefore viewed with an extreme caution.

## Introduction

The term ‘polycystic ovarian syndrome’ (PCOS) represents a heterogeneous and multifaceted entity characterised by hyperandrogenism and/or ovulatory dysfunction. It is also the most common endocrinopathy of women of reproductive age (1, 2). According to the Rotterdam criteria (2003) (3), a diagnosis of PCOS can be established when at least two out of three criteria are present (oligo-/anovulation, clinical hyperandrogenism or biochemical hyperandrogenaemia and polycystic ovaries) on condition that other causes of oligo-/anovulation or hyperandrogenism/hyperandrogenaemia (hyperprolactinaemia, Cushing’s syndrome, congenital adrenal hyperplasia, premature ovarian failure, hypothalamic/pituitary disease, etc.) have been ruled out.

Though it is widely accepted that PCOS is characterised by insulin resistance (4), there is no consensus, either regarding the best method of assessment of insulin resistance (IR) in PCOS, nor in terms of the utility of such assessment for subsequent clinical management, such as indications for treatment with insulin-sensitising agents (5). It is also well recognised that an euglycaemic–hyperinsulinaemic clamp technique (6), regarded as the golden standard for assessment of IR, is too laborious and complicated for use outside research settings. Furthermore, there are also limitations for the use of surrogate models for IR assessment, where it is not possible to stipulate, which method can be regarded as optimal (7).

The aim of the study was to compare most commonly used indices of IR (HOMA-IR and QUICKI) with the McAuley index that utilises fasting triglycerides, rather than fasting glucose concentrations. The authors of the latter model (8), claim that in the multivariate model, inclusion of fasting triglycerides increases sensitivity of the model to assess IR even better than HOMA, fasting insulin or fasting insulin-to-glucose ratio.

The second aim of the study was to compare the above mentioned indices of IR derived from fasting values (insulin plus glucose/triglycerides) with indices of IR derived from glucose and insulin measurements during 75 g oral glucose tolerance test (OGTT), such as Insulin Resistance (Belfiore) Index (IRI) (9), Matsuda index (10) and Stumvoll index (11).

## Subjects and methods

The study included 478 women aged 24.75 ± 8.05 years (mean ± s.d.), body mass index (BMI) 27.27 ± 7.18 kg/m^2^ who underwent investigations for irregular periods, hirsutism or biochemical hyperandrogenism in the Department of Endocrinology and Metabolic Diseases, The Medical University of Lodz and The Polish Mother’s Memorial Hospital Research Institute in Lodz, Poland (between 2013 and 2016). A diagnosis of PCOS was established according to the Rotterdam consensus criteria (3). All patients were subjected to an identical investigation protocol that included hormonal assessment (TSH, free T_4_ and free T_3_, prolactin, total testosterone, androstenedione, DHEAS, 17-hydroxy-progesterone, cortisol after 1 mg overnight dexamethasone suppression test, fasting blood lipids and intravaginal pelvic ultrasound). All subjects also underwent glucose and insulin measurements during 75 g OGTT, where measurements were performed at 0, 60 and 120 min.

If clinically indicated, additional tests (such as IGF-I, growth hormone during OGTT, 17-hydroxy-progesterone measurements after 250 µg of intravenous Synacthen, 24-h prolactin secretion profiles) were performed. All investigations were performed in the early follicular phase of either a spontaneous cycle or after induction of the menstrual bleeding with a progestagen (usually dydrogesterone (Duphaston) 10 mg twice a day for ten days).

Insulin resistance index (IRI) was calculated from changes of glycaemia and insulinaemia during a 75 g oral glucose tolerance test (OGTT) according to the method described by Belfiore and coworkers (9). IRI was calculated through the formula: ISI_(Gly)_ = 2/(1/(INSp × GLYp)) + 1, where INSp and GLYp are the measured insulin and glycaemic areas. In normal subjects, ISI(gly) are always around 1, with maximal variations between 0 and 2. This method is based on changes of glycaemia and insulinaemia during OGTT. According to the same authors, the assessment of free fatty acids (FFA) during OGTT is equally effective for the purpose of calculation of the IRI (9).

HOMA-IR index was calculated according to the formula: HOMA-IR = (fasting glucose) (mmol/L) × (fasting insulin) (µU/mL)/22.5) (12). QUICKI index was calculated according to the formula: QUICKI = 1/(log(I_0_) + log(G_0_)), where I_0_ denotes fasting insulin and G_0_ denotes fasting glucose (13).

McAuley index was calculated according to the formula: Mffm/I = e (2.63 – 0.28 ln (I_0_) – 031 ln (TAG_0_)), where TAG_0_ denotes fasting triglyceride concentrations (8).

Matsuda index (10) was calculated according to formula:


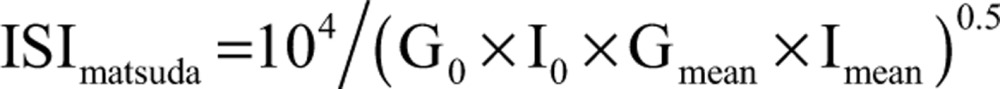


where: I_0_, fasting plasma insulin concentration (IU/L); G_0_, fasting plasma glucose concentration (mg/dL); G_mean_, mean plasma glucose concentration during OGTT (mg/dL); I_mean_, mean plasma insulin concentration during OGTT (U/L).

As in fact there are several formulae used to calculate Stumvoll index (11), we have chosen the most commonly used two formulae:


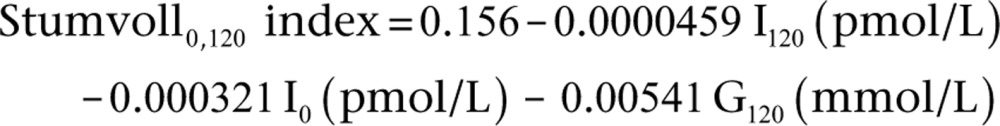


where: I_0_, fasting insulin (pmol/L); I_120_, insulin concentration at 120 min of OGTT (pmol/L); G_120_, glucose concentrations at 120 min of OGTT (mmol/L).

As inclusion of parameters, such as age and BMI, in our opinion, could enrich analysed models, based almost exclusively on glucose and insulin, then we have decided to include into our analysis also a formula for the Stumvoll index that involves few measurements during OGTT, but incorporates demographic data, such as age and BMI into the model:


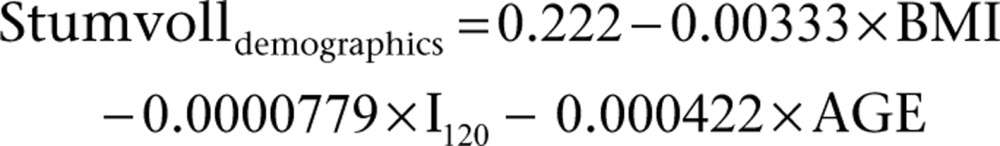


where: I_120_, insulin concentration at 120 min of OGTT (pmol/L).

As patients previously diagnosed with type 2 diabetes according to high fasting blood glucose criterion (glucose concentrations >7.0 mmol/L) do not require an OGTT to confirm a diagnosis of diabetes, then they were not included into the study.

Statistical analysis was performed by the means of MedClac software, version 16.4.3.

Clinical and hormonal characteristics of subjects participating in the study are presented in the study are presented in Table 1. The study was approved by the Ethics Committee of the Polish Mother’s Memorial Hospital–Research Institute.

**Table 1:** Clinical, biochemical and hormonal characteristics of subjects participating in the study (*n* = 478).

	Mean	s.d.
Age (years)	24.75	8.05
BMI (kg/m^2^)	27.27	7.18
Total cholesterol (mg/dL)	170	34.39
LDL cholesterol (mg/dL)	105	57.64
HDL cholesterol (mg/dL)	52	16.25
Triglycerides (mg/dL)	104	60.64
17-Hydroxy-progesterone (ng/mL)	1.09	0.62
Oestradiol (pg/mL)	69	83.80
Free T_4_ (ng/dL)	1.25	0.32
TSH (IU/L)	2.08	1.19
Glucose (mmol/L)	4.57	0.45
Insulin (IU/mL)	12	9.28
HOMA-IR	2.6695	2.0990
QUICKI	0.6123	0.1150
McAuley index	7.2951	1.9786
IRI (Belfiore)	1.19	0.36
Matsuda index	5.9304	4.0530
Stumvoll 0–120 index	0.07200	0.04793
Stumvoll with demographics index	0.07932	0.05875
Androstenedione (ng/mL)	3.95	1.73
DHEAS (µg/dL)	306	132.19
Total testosterone (µg/mL)	0.53	0.23

Consent has been obtained from each patient or subject after full explanation of the purpose and nature of all procedures used.

## Results

During OGTT, 19 patients (3.97%) were found to have impaired fasting glucose (i.e. glucose concentrations 5.56–7.0 mmol/L), 42 patients (8.78%) were found to have impaired glucose tolerance (i.e. glucose concentration 7.0–11.1 mmol/L at 120 min of OGTT), while 5 patients (1.04%) were found to have frank diabetes (glucose concentrations >11.1 mmol/L at 120 min of OGTT). Four of these patients (0.8%) were also found to have simultaneously impaired fasting glucose. Coexistence of both impaired fasting glucose and impaired glucose tolerance was observed in eight (1.67%) patients.

Percentile distribution of IR indices calculated according the HOMA, IRI, QUICKI, McAuley index, Matsuda index and Stumvoll_0,120_ and Stumvoll_demographics_ models is presented in Table 2.

**Table 2:** Percentile distribution of insulin resistance indices in a group of 478 women with polycystic ovary syndrome.

Percentiles	HOMA (95% CI)		IRI (Belfiore) (95% CI)		QUICKI (95% CI)		McAuley (95% CI)		Matsuda (95% CI)		Stumvoll 0–120 (95% CI)		Stumvoll with demographics (95% CI)	
2.5	0.63	0.42–0.72	0.27	0.00–0.38	0.42	0.40–0.44	3.9071	3.3684–4.3353	1.14	0.83–1.42	−0.06	−0.13 to −0.02	−0.06	−0.15 to −0.03
5	0.78	0.66–0.87	0.47	0.33–0.55	0.40	0.39–0.41	4.4577	4.0308–4.6674	1.47	1.16–1.75	−0.01	−0.059 to 0.004	−0.03	−0.056 to −0.015
10	0.97	0.87–1.01	0.65	0.57–0.70	0.385	0.38–0.39	4.9480	4.6884–5.1908	1.92	1.65–2.17	0.017	0.0003–0.028	0.006	–0.016 to 0.014
25	1.42	1.30–1.54	0.89	0.83–0.92	0.36	0.35–0.37	5.7902	5.5725–6.0672	3.12	2.65–3.37	0.057	0.048–0.065	0.052	0.037–0.064
75	3.25	2.94–3.51	1.46	1.43–1.51	0.32	0.31–0.33	8.6044	8.3731–8.8564	7.66	6.92–8.48	0.10	0.099–0.105	0.12	0.11–0.12
90	4.60	4.25–5.77	1.67	1.63–1.70	0.30	0.29–0.31	10.0723	9.7055–10.3394	11.46	10.13–12.30	0.111	0.11–0.115	0.131	0.12–0.13
95	6.68	5.84–7.70	1.74	1.70–1.79	0.29	0.28–0.30	10.6709	10.347–11.1762	14.03	12.00–16.75	0.118	0.114–0.119	0.137	0.13–0.14
97.5	8.53	7.23–9.82	1.82	1.76–1.85	0.28	0.27–0.29	11.6127	10.853–12.0542	17.23	14.61–20.58	0.12	0.118–0.123	0.142	0.13–0.14

CI, confidence interval.

**Figure 1:**
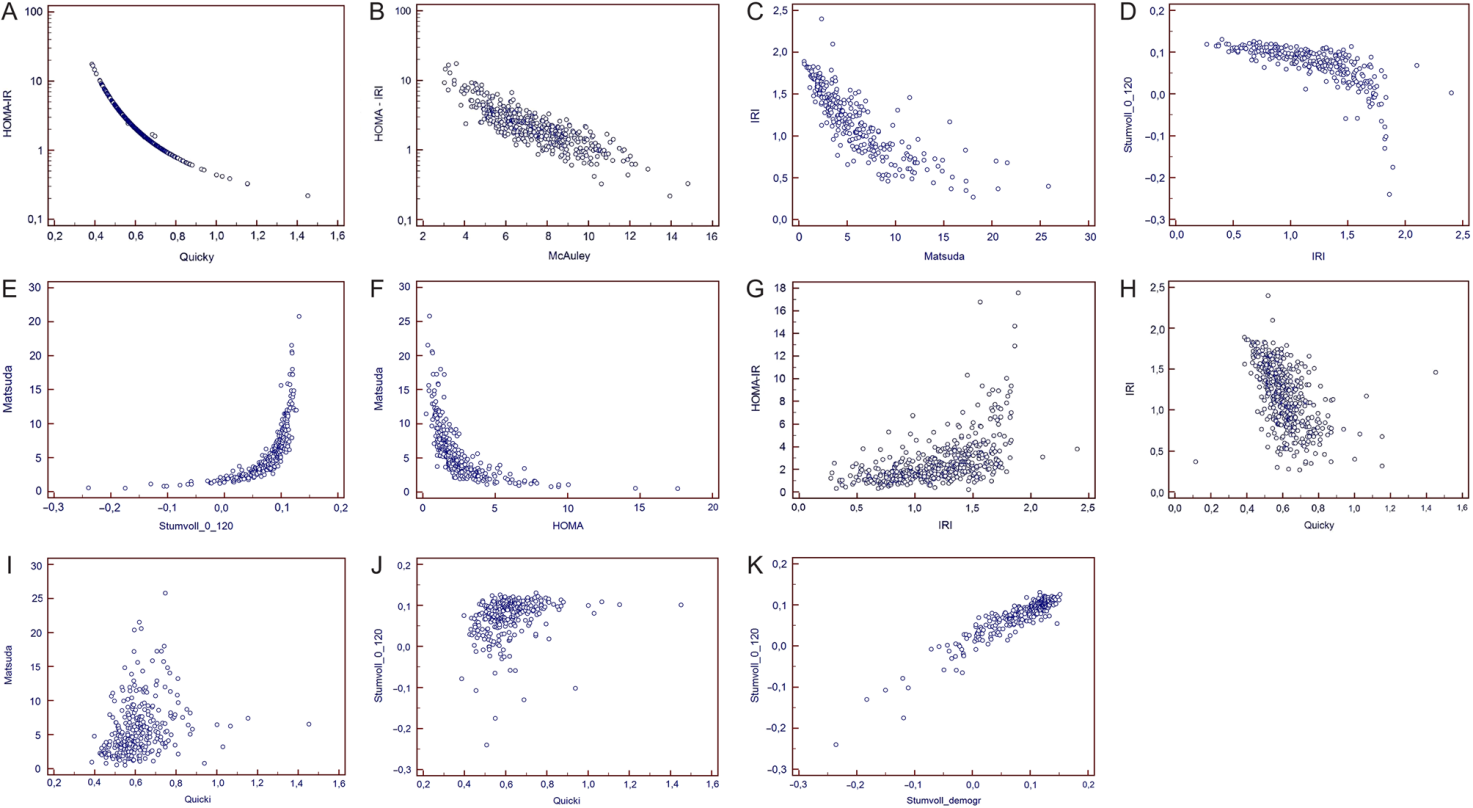
(A) Correlation between HOMA-IR and QUICKI (*r* = −0.999, *P* < 0.001). (B) Correlation between HOMA-IR and McAuley index (*r* = −0.849, *P* < 0.001). (C) Correlation between Insulin Resistance (Belfiore) Index (IRI) and Matsuda index (*r* = −0.876, *P* < 0.001). (D) Correlation between Insulin Resistance (Belfiore) Index (IRI) and Stumvoll_0,120_, (*r* = −0.808, *P* < 0.001). (E) Correlation between Stumvoll_0,120_ and Matsuda index (*r* = 0.947, *P* < 0.001). (F) Correlation between Matsuda index and HOMA-IR (*r* = −0.881, *P* < 0.001). (G) Correlation between HOMA-IR and Insulin Resistance (Belfiore) Index (IRI) (*r* = 0.582, *P* < 0.001). (H) Correlation between Insulin Resistance (Belfiore) Index (IRI) and QUICKI (*r* = −0.580, *P* < 0.001). (I) Correlation between Matsuda index and QUICKI (*r* = 0.386, *P* < 0.001). (J) Correlation between Stumvoll_0,120_ and QUICKI (*r* = 0.410, *P* < 0.001). (K) Correlation between Stumvoll_0,120_ and Stumvoll_demographics_ (*r* = 0.874, *P* < 0.001).

Spearman rank correlations between insulin resistance indices are presented in Table 3. There was a very good correlation (*P* < 0.001) between IR indices based on fasting values of glucose and insulin and/or triglycerides, e.g. HOMA vs QUICKI, *r* = −0.999, Fig. 1A, HOMA-IR vs McAuley, *r* = −0.849 (Fig. 1B and Table 3). There was also a very good and significant correlation between OGTT-derived IR indices (*P* < 0.001), for instance, IRI vs Matsuda index, *r* = −0.876 (Fig. 1C), IRI vs Stumvoll_0,120_ (Fig. 1D), *r* = −0.808, Stumvoll_0,120_, vs Matsuda index, *r* = 0.947 (Fig. 1E). In contrast, correlation between IR indices derived from OGTT and fasting values was highly variable, ranging from relatively good correlation between Matsuda index and HOMA-IR (*r* = −0.881) (Fig. 1F), through moderate correlation between HOMA-IR and IRI (0.582) (Fig. 1G) or IRI vs QUICKI (*r* = −0.580) (Fig. 1H), up to rather weak correlation between Matsuda index and QUICKI (*r* = 0.386) (Fig. 1I) or between Stumvoll_0,120_ and QUICKI (*r* = 0.410) (Fig. 1J). Correlation between Stumvoll_demographics_ index, that involves data such as age and BMI, but only single insulin concentration at 120 min of OGTT and other IR indices also ranged from rather weak correlation with QUICKI (*r* = 0.439), to good correlation with IRI (*r* = −0.773) and Matsuda index (*r* = 0.856) (Table 3). Interestingly, correlation between two types of Stumvoll indices (i.e. Stumvoll_0,120_ that includes insulin concentrations at 0 and 120 min of OGTT, and glucose at 120 min OGTT) vs Stumvoll_demographics_ (that utilises age, BMI, single insulin concentration at 120 min of OGTT and no glucose), was good (*r* = 0.874) (Fig. 1K), but not as perfect as correlation between HOMA-IR and QUICKI (*r* = −0.999) or Matsuda and Stumvoll_0,120_ (*r* = 0.947) (Table 3). There was a weak, but significant correlation of all IR indices with serum total testosterone concentrations (Table 4).

**Table 3:** Spearman rank correlation between insulin resistance indices (*P* < 0.001).

	IRI	QUICKi	McAuley	Matsuda	Stumvoll_0,120_	Stumvoll demogr.
HOMA-IR	0.582	−0.999	−0.849	−0.881	−0.832	−0.694
IRI (Belfiore)	–	−0.580	−0.614	−0.876	−0.808	−0.773
QUICKI	–	–	0.850	0.386	0.410	0.439
McAuley	–	–	–	0.551	0.569	0.618
Matsuda	–	–	–	–	0.947	0.856
Stumvoll_0,120_	–	–	–	–	–	0.874

**Table 4:** Spearman correlation coefficients of serum androgens with insulin resistance indices.

	Total testosterone	Androstenedione	DHEA-S
HOMA	0.155	0.124	0.0327
IRI (Belfiore)	0.139	0.0536	0.0825
QUICKI	−0.152	−0.124	−0.0382
McAuley	−0.150	−0.122	−0.0358
Matsuda	−0.149	−0.110	−0.0448
Stumvoll 0–120	−0.182	−0.128	−0.0625
Stumvoll demogr.	−0.201	−0.146	−0.0541

Significant correlations (*P* < 0.05) are marked in red.

**Figure 2:**
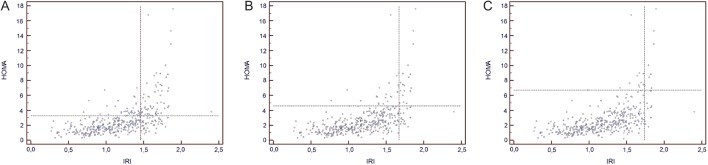
(A) Concordance and discordance between insulin resistance indices assessed by HOMA-IR and Insulin Resistance (Belfiore) Index at 75th percentile of data distribution. (B) Concordance and discordance between insulin resistance indices assessed by HOMA-IR and Insulin Resistance (Belfiore) Index at 90th percentile of data distribution. (C) Concordance and discordance between insulin resistance indices assessed by HOMA-IR and Insulin Resistance (Belfiore) Index at 95th percentile of data distribution.

In further analysis, we have assessed concordance and discordance between selected IR indices. Due to a strong correlation between HOMA-IR and QUICKI indices (*r* = −0.999), only comparison between HOMA-IR and IRI was used for further assessment. We have also chosen comparison between HOMA-IR and IRI indices, as these indices are routinely used for assessment of IR in our department. Further analysis (Fig. 2A, B and Tables 5, 6) revealed that a significant number of patients would be differently classified in terms of percentile distribution, according to the method applied. Hence, at 75th percentile, out of 132 patients found to be above 75th percentile for IRI, only 70 (53%) would be concomitantly found to be above 75th percentile according to HOMA-IR. The same persisted for 90th percentile, where only 44% of patients found to be above 90th percentile for IRI, was simultaneously above 90th percentile for HOMA-IR (Fig. 2B and Table 6). These mentioned discrepancies tended to persist even at extremes of IR spectrum (i.e. above 95th percentile – Fig. 2C and Table 7). Interestingly, the above discrepancies were even amplified, where IRI was compared to the data obtained from McAuley index, where 121/126 (96%), and 52/53 (98%) women with IRI above 75th, and 90th percentile had the value of McAuley index below 75th and 90th percentiles, respectively (Tables 8 and 9). The above mentioned discordance also persisted for values above 95th percentile (Table 10).

**Table 5:** Concordance and discordance between insulin resistance indices assessed by HOMA-IR and Insulin Resistance (Belfiore) Index at 75th percentile of data distribution.

	HOMA-IR		
	≤3.25	>3.25	Total
IRI			
≤1.46	295 (85%)	51 (15%)	346 (72.4%)
>1.46	62 (47%)	70 (53%)	132 (27.6%)
Total	357 (74.7%)	121 (25.3%)	478 (100%)

**Table 6:** Concordance and discordance between insulin resistance indices assessed by HOMA-IR and Insulin Resistance (Belfiore) Index at 90th percentile of data distribution.

	HOMA-IR		
	≤4.6	>4.6	Total
IRI			
≤1.67	400 (94%)	24 (6%)	424 (88.7%)
>1.67	30 (56%)	24 (44%)	54 (11.3%)
Total	430 (90.0%)	48 (10.0%)	478 (100%)

**Table 7:** Concordance and discordance between insulin resistance indices assessed by HOMA-IR and Insulin Resistance (Belfiore) Index at 95th percentile of data distribution.

	HOMA-IR		
	≤6.68	>6.68	Total
IRI			
≤1.74	432 (95.8%)	19 (4.2%)	451 (94.4%)
>1.74	23 (85.2%)	4 (14.8%)	54 (11.3%)
Total	455 (95.2%)	23 (4.8%)	478 (100%)

**Table 8:** Concordance and discordance between insulin resistance indices assessed by McAuley index at 75th percentile of data distribution.

	McAuley		
	≤8.6	>8.6	Total
IRI			
≤1.46	253 (71.1%)	103 (28.9%)	356 (73.9%)
>1.46	121 (96.0%)	5 (4.0%)	126 (26.1%)
Total	374 (77.6%)	108 (22.4%)	482 (100%)

**Table 9:** Concordance and discordance between insulin resistance indices assessed by McAuley index at 90th percentile of data distribution.

	McAuley		
	≤10.07	>10.07	Total
IRI			
≤1.67	387 (90.2%)	42 (9.8%)	429 (89.0%)
>1.67	52 (98.1%)	1 (1.9%)	53 (11.0%)
Total	439 (91.1%)	43 (8.9%)	482 (100%)

**Table 10:** Concordance and discordance between insulin resistance indices assessed by McAuley index at 95th percentile of data distribution.

	McAuley		
	≤10.67	>10.67	Total
IRI			
≤1.74	432 (95.6%)	20 (4.4%)	452 (93.8%)
>1.74	29 (96.7%)	1 (3.3%)	30 (6.2%)
Total	461 (95.6%)	21 (4.4%)	482 (100%)

## Discussion

This study, based on analysis of one of the largest group of women with PCOS, diagnosed in a single centre, according to an identical protocol, leads to three main conclusions. First, there is a very good correlation between indices of IR not only based on fasting glucose and insulin (i.e. HOMA and QUICKI), but also with an McAuley index that utilises fasting triglyceride concentrations instead of fasting glucose. Hence, there is an implication that fasting triglyceride concentrations can be safely used to assess IR in PCOS, instead of fasting glucose. In previous study by Garg and coworkers (14), McAuley index was found to have the greatest specificity in large (*n* = 695) group of Indian adolescents, with an excellent correlation with HOMA-IR (*r* ^2^ = 0.965).

Second, correlation between indices of IR based of fasting values (HOMA-IR, QUICKI and McAuley index) is, however, very variable, when compared to assessment of IR by the means of methods based on measurements of glucose and insulin during OGTT, i.e. Insulin Resistance (Belfiore) Index, two variations of Stumvoll index (i.e. Stumvoll_0,120_ and Stumvoll_demographics_) and Matsuda index. This is particularly relevant for QUICKI (i.e. an index involving logarithmic transformation of analysed data), where correlation coefficient might be as low as *r* = 0.386 (QUICKI vs Matsuda index). Correlation coefficients are slightly better, but still rather moderate, for McAuley index, while the greatest variation involves HOMA-IR, that ranges from *r* = 0.582 for correlation with IRI, though *r* = −0.694, with Stumvoll_demographics_, up to a very good correlation with Stumvoll_0,120_ and Matsuda indices (*r* = −0.832, and *r* = −0.881, respectively – Table 3). On the other hand, there is quite good correlation between all OGTT-derived IR indices (i.e. IRI, Matsuda index, Stumvoll_0,120_) with slightly weaker correlation with Stumvoll_demographics_, that, however, includes only a single OGTT-derived parameter (insulin at 120 min). Despite very good correlation between HOMA-IR and Matsuda index, in our opinion, some caution is required before we classify the Matsuda index as the best to tool to investigate IR in PCOS. This is because in PCOS patients (15) (*n* = 100) correlation of the Matsuda index with the clamp technique (*r* = 0.668) was very similar to Belfiore_area_ index (*r* = 0.645), while the authors state that: ‘The presence of a high correlation coefficient does not mean that these indexes have the best predictive performance in diagnosing insulin resistance, because of the presence of many borderline values.’ Furthermore, correlation is much weaker with other indices derived from fasting values, i.e. McAuley index, and particularly for QUICKI. There are also data that correlation between HOMA-IR and insulin concentrations during OGTT in women with PCOS is relatively modest (for instance *r* = 0.42, and *r* = 0.52, at 60 and 120 min of OGTT respectively) (16). Furthermore, it is known that IR indices derived from fasting glucose and insulin predominantly reflect hepatic rather than peripheral insulin sensitivity that is more reflected by indices that are based on glucose and insulin during OGTT (17, 18).

As correlation between IR indices based on fasting vs OGTT-derived data are highly variable even if identical clinical data (e.g. fasting glucose and insulin) are included into the mathematical model (see HOMA-IR and QUICKI); hence, in our opinion, it is virtually impossible to select ‘the best’ surrogate method for the assessment of insulin resistance in women with PCOS.

In our study, we observed a significant, but relatively weak correlation between all analysed IR indices and total testosterone. Indeed more IR subjects seem to have higher testosterone/dihydrotestosterone ratio, and significant, though not particularly strong correlation with HOMA-IR and QUICKI was reported before (19). To the best of our knowledge, this is, however, the first study, where six different IR indices were correlated with serum androgens in such a large group of women with PCOS. Interestingly, we did not observe a correlation of IR indices with DHEAS that was reported by Brennan and coworkers (20), though those authors used only HOMA-IR model for their assessment.

From clinical perspective, it is important that significant discrepancies between the methods based on fasting values and OGTT-derived values seem to persist even at the extremes of insulin sensitivity spectrum, i.e. when analysed according to percentiles of data distribution. Hence, a significant number of women classified as most insulin resistant according to one method/s (e.g. based on OGTT-derived data), might be found to be less (or more) insulin resistant according to a different method (e.g. based on fasting data), regardless of a percentile used as a cut-off point. Thus, if a 75th percentile is used, then 47% of women found to be insulin resistant by IRI, would fall below 75th percentile for HOMA. Due to an excellent correlation between HOMA-IR, QUICKI indices, we can assume that the same situation would apply, if QUICKI index were substituted for HOMA-IR. The same applies to 90th percentile, as well as to 95th percentile of data distribution. Interestingly, the opposite situation, i.e. the number of women found to be more insulin resistant according to HOMA-IR than IRI, appears to be less frequent (15%, and 6%, respectively, for 75th and 90th percentile). Given only moderate correlation between McAuley index and OGTT-derived indices (Matsuda, IRI (Belfiore) and Stumvoll) assessment IR by the means of an McAuley index should not be extrapolated to IR assessment based on glucose and insulin measurements during OGTT, as discrepancies are even greater at upper extremes of IR percentile distributions (Tables 8, 9 and 10).

The last, but not the least, we can state, even in the absence of a control group, that women with PCOS seem to be more insulin resistant than the general population. For instance, 75th percentile for HOMA-IR is 3.25 for our population of women with PCOS, while 75th percentile for the Polish population of Krakow (Poland) was reported as 2.1 (21), and 2.29 for the Czech population (22). Most quoted cut-off point for the 90th percentile for HOMA-IR is 3.8 (23), though precise calculations, also for the Spanish population suggested a cut-off for HOMA-IR of 3.46 (24), at 90th percentile of data distribution. For comparison, a 90th percentile for HOMA-IR for our PCOS patients equalled 4.6. The same applies for the Insulin Resistance (Belfiore) Index, where cut-off points for the 75th and 90th percentile were 1.46 and 1.67, respectively, while the quoted cut-off point for this index (no percentile specified) is 1.27 (25). Mean values of HOMA-IR and QUICKI in our study, are, however, similar to data of Christodoulopoulou and coworkers (26), based on a group of 309 Greek women with PCOS. It must be remembered, however, that our data, though based on a large group of women with PCOS, have been obtained from an almost entirely Caucasian population, while percentile distribution for IR indices may be different, if derived from other (e.g. Asian) populations (27).

Finally, the results of our study should be interpreted in view of potential utility of IR assessment in women with PCOS. The issue of insulin resistance in PCOS, though seemingly obvious, is indeed highly problematic, when supposed to be transformed from a theoretical concept into a clinical application. In a seminal paper by Dunaif and coworkers (28), IR in PCOS was assessed by the means of euglycaemic glucose-clamp technique in a relatively small group of women, i.e. in nineteen obese and ten non-obese women with PCOS vs eleven obese and eight non-obese controls. The authors concluded that IR was apparent not in terms of surpassing of any predefined cut-off point, based on selected surrogate IR indices, but as a decreased insulin sensitivity, determined by the rate of glucose infusion during the clamp, in comparison to BMI-matched non-PCOS peers. Hence, application of any surrogate insulin resistance indices must be viewed with an extreme caution, as suggested by Diamanti-Kandarakis and coworkers (29). Unfortunately, clinical application of the clamp studies apart from technical difficulties, is confounded by small sample sizes, missing data and the lack of Rotterdam criteria phenotype reporting, thus limiting the validity of available evidence (30). On the other hand, application of various cut-off points for both fasting and OGTT-derived IR indices results in wide variations in estimated prevalence of IR in PCOS women ranging from 12.2 to 60.5% (31), as it was confirmed by our concordance/discordance analysis of selected IR indices. It should also be mentioned that some studies have cast doubt on previously assumed good correlation of data obtained from surrogate IR indices and data obtained from an euglycaemic clamp technique, both for fasting glucose and insulin models (32) and for methods based on glucose and insulin during OGTT (33). In the latter case, some authors raise the issue that indices derived from OGTT could be subjected to many confounders (34). Correlation of IR indices with the clamp method is also influenced by BMI, for instance according to Ruige and coworkers (35), Spearman correlation coefficient between clamp method and HOMA, QUICKI and McAuley index is 0.47 for subjects with BMI >25 kg/m^2^, but drops to 0.33 (HOMA and QUICKI) and even to 0.22 for McAuley index, for subjects with BMI <25 kg/m^2^. The effects of BMI are also demonstrated in a study by Chrenova and coworkers (36) (*n* = 119), where the authors compared several IR indices (both based on fasting and OGTT-derived data) in terms how they discriminate IR resistance between lean and obese. They demonstrated that QUICKI index and Stumvoll_demographics_ offer the least contrast in IR between lean and obese, with much greater contrast obtained by Matsuda and HOMA-IR indices (Stumvoll_0,120_ performing somewhere in the middle). On the other hand, while analysing the spread of individual values from the mean value obtained by the chosen method, the best performance was obtained for Stumvoll_0,120_, and Stumvoll_demographics_ indices, but only for lean subjects. In obese individuals, Stumvoll_0,120_, performed reasonably well, contrasting with Stumvoll_demographics_, that offered the least spread. Ciampelli and coworkers (15) demonstrated that different IR indices performed best in PCOS vs postmenopausal women. Hence, a performance of a chosen model for IR assessment may be widely different depending on the characteristics of the studied population, and it is virtually impossible to choose a model that would perform equally well in all populations, regardless of their age, BMI and racial background.

In summary, analysis of our data, based on a large number of women with PCOS, demonstrated that fasting triglycerides can be safely used instead of fasting glucose for assessment of insulin resistance, when compared to methods utilising fasting glucose and insulin. On the other hand, application of methods based on glucose and insulin measurements during OGTT vs methods based entirely on fasting data yields discrepant results in terms of severity of insulin resistance in a significant number of women with PCOS, with a wide variation of correlation coefficients between various methods. Hence, women classified as insulin resistant by one method, might not be equally insulin resistant if analysed by another method. Therefore, in our opinion, currently it is not possible to select either a universal ‘cut-off’ point in order to define insulin resistance, nor to define ‘the best’ method of assessment of insulin resistance, for the purpose of clinical practice.

## Declaration of interest

The authors declare that there is no conflict of interest that could be perceived as prejudicing the impartiality of the research reported.

## Funding

The study was supported by the statutory funds from the Medical University of Lodz (503/1-107-03/503-11-001) and Polish Mother’s Memorial Hospital – Research Institute, Lodz, Poland.

## References

[1] Azziz R Woods KS Reyna R Key TJ Knochenhauer ES Yildiz BO. The prevalence and features of the polycystic ovary syndrome in an unselected population. Journal of Clinical Endocrinology and Metabolism 2004 89 2745–2749. (10.1210/jc.2003-032046) 15181052

[2] Welt CK Carmina E. Clinical review: lifecycle of polycystic ovary syndrome (PCOS): from in utero to menopause. Journal of Clinical Endocrinology and Metabolism 2013 98 4629–4638. (10.1210/jc.2013-2375) 24064685 PMC3849665

[3] Rotterdam ESHRE/ASRM-Sponsored PCOS Consensus Workshop Group. Revised 2003 consensus on diagnostic criteria and long-term health risks related to polycystic ovary syndrome (PCOS). Human Reproduction 2004 19 41–47. (10.1093/humrep/deh098) 14688154

[4] Macut D Bjekic-Macut J Rahelic D Doknic M. Insulin and the polycystic ovary syndrome. Diabetes Research and Clinical Practice 2017 130 163–170. (10.1016/j.diabres.2017.06.011) 28646699

[5] Sam S Ehrmann DA. Metformin therapy for the reproductive and metabolic consequences of polycystic ovary syndrome. Diabetologia 2017 60 1656–1661. (10.1007/s00125-017-4306-3) 28770330

[6] DeFronzo RA Tobin JD Andres R. Glucose clamp technique: a method for quantifying insulin secretion and resistance. American Journal of Physiology 1979 237 E214–E223. (10.1152/ajpendo.1979-237-3) 382871

[7] Szosland K Lewinski A. In quest for method of insulin resistance assessment in everyday clinical practice-insulin resistance indices. Diabetes and Metabolic Syndrome 2016 10 (Supplement 1) S120–S125. (10.1016/j.dsx.2015.10.007) 26616342

[8] McAuley KA Williams SM Mann JI Walker RJ Lewis-Barkned NJ Templer LA Duncan AW. Diagnosing insulin resistance in the general population. Diabetes Care 2001 24 460–464. (10.2337/diacare.24.3.460) 11289468

[9] Belfiore F Iannello S Volpicelli G. Insulin sensitivity indices calculated from basal and OGTT-induced insulin, glucose, and FFA levels. Molecular Genetics and Metabolism 1998 63 134–141. (10.1006/mgme.1997.2658). 9562967

[10] Matsuda M DeFronzo RA. Insulin sensitivity indices obtained from oral glucose tolerance testing: comparison with the euglycemic insulin clamp. Diabetes Care 1999 22 1462–1470. (10.2337/diacare.22.9.1462). 10480510

[11] Stumvoll M Mitrakou A Pimenta W Jenssen T Yki-Järvinen H Van Haeften T Renn W Gerich J. Use of the oral glucose tolerance test to assess insulin release and insulin sensitivity. Diabetes Care 2000 23 295–301. (10.2337/diacare.23.3.295) 10868854

[12] Matthews DR Hosker JP Rudenski AS Naylor BA Treacher DF Turner RC. Homeostasis model assessment: insulin resistance and beta-cell function from fasting plasma glucose and insulin concentrations in man. Diabetologia 1985 28 412–419. (10.1007/BF00280883) 3899825

[13] Katz A Nambi SS Mather K Baron AD Follmann DA Sullivan G Quon MJ. Quantitative insulin sensitivity check index: a simple, accurate method for assessing insulin sensitivity in humans. Journal of Clinical Endocrinology and Metabolism 2000 85 2402–2410. (10.1210/jcem.85.7.6661). 10902785

[14] Garg MK Tandon N Marwaha RK Singh Y. Evaluation of surrogate markers for insulin resistance for defining metabolic syndrome in urban Indian adolescents. Indian Pediatrics 2014 51 279–284. (10.1007/s13312-014-0401-4) 24825264

[15] Ciampelli M Leoni F Cucinelli F Mancuso S Panunzi S De Gaetano A Lanzone A. Assessment of insulin sensitivity from measurements in the fasting state and during an oral glucose tolerance test in polycystic ovary syndrome and menopausal patients. Journal of Clinical Endocrinology and Metabolism 2005 90 1398–1406. (10.1210/jc.2004-0410) 15598698

[16] Jovanovska-Mishevska S Atanasova-Boshku A Bitoska I Ahmeti I Todorova B Pemovska G Milenkovic T Krstevska B. Indexes of insulin resistance in hyperinsulinemic polycystic ovary syndrome in a Macedonian cohort of women of reproductive age: a cross-sectional study. Open Access Macedonian Journal of Medical Sciences 2016 4 607–612. (10.3889/oamjms.2016.107) 28028399 PMC5175507

[17] Hoffman RP. Indices of insulin action calculated from fasting glucose and insulin reflect hepatic, not peripheral, insulin sensitivity in African-American and Caucasian adolescents. Pediatric Diabetes 2008 9 57–61. (10.1111/j.1399-5448.2007.00350.x) 18221434

[18] Singh B Saxena A. Surrogate markers of insulin resistance: a review. World Journal of Diabetes 2010 1 36–47. (10.4239/wjd.v1.i2.36) 21537426 PMC3083884

[19] Münzker J Hofer D Trummer C Ulbing M Harger A Pieber T Owen L Keevil B Brabant G Lerchbaum E, Testosterone to dihydrotestosterone ratio as a new biomarker for an adverse metabolic phenotype in the polycystic ovary syndrome. Journal of Clinical Endocrinology and Metabolism 2015 100 653–660. (10.1210/jc.2014-2523) 25387259

[20] Brennan K Huang A Azziz R. Dehydroepiandrosterone sulfate and insulin resistance in patients with polycystic ovary syndrome. Fertility and Sterility 2009 91 1848–1852. (10.1016/j.fertnstert.2008.02.101) 18439591 PMC2691796

[21] Szurkowska M Szafraniec K Gilis-Januszewska A Szybiński Z Huszno B. Insulin resistance indices in population-based study and their predictive value in defining metabolic syndrome. Przegląd Epidemiologiczny 2005 59 743–751. 16433317

[22] Radikova Z Koska J Huckova M Ksinantova L Imrich R Vigas M Trnovec T Langer P Sebokova E Klimes I., Insulin sensitivity indices: a proposal of cut-off points for simple identification of insulin-resistant subjects. Experimental and Clinical Endocrinology and Diabetes 2006 114 249–256. (10.1055/s-2006-924233) 16804799

[23] Martizez-Larrad MT Corbaton Anchuelo A Del Prado N Ibarra Rueda JM Gabriel R Serrano-Rios M. Profile of individuals who are metabolically healthy obese using different definition criteria. A population-based analysis in the Spanish population. PLoS ONE 2014 9 e106641 (10.1371/journal.pone.0106641) 25198070 PMC4157807

[24] Gayoso-Diz P Otero-González A Rodriguez-Alvarez MX Gude F García F De Francisco A Quintela AG. Insulin resistance (HOMA-IR) cut-off values and the metabolic syndrome in a general adult population: effect of gender and age: EPIRCE cross-sectional study. BMC Endocrine Disorders 2013 13 47 (10.1186/1472-6823-13-47). 24131857 PMC4016563

[25] Gutch M Kumar S Razi SM Gupta JJ Gupta A. Assessment of insulin sensitivity/resistance. Indian Journal of Endocrinology and Metabolism 2015 19 160–164. (10.4103/2230-8210.146874) 25593845 PMC4287763

[26] Christodoulopoulou V Trakakis E Pergialiotis V Peppa M Chrelias Ch Kassanos D Papantonoiou N. Clinical and biochemical characteristics in PCOS women with menstrual abnormalities. Journal of Family and Reproductive Health 2016 10 184–190. 28546817 PMC5440817

[27] Wijeyarte CN Balen AH Barth JH Belchetz PE. Clinical manifestations and insulin resistance (IR) in polycystic ovary syndrome (PCOS) among South Asians and Caucasians: is there a difference? Clinical Endocrinology 2002 57 343–350. (10.1046/j.1365-2265.2002.01603.x) 12201826

[28] Dunaif A Segal KR Futterweit W Dobrjansky A. Profound peripheral insulin resistance, independent of obesity, in polycystic ovary syndrome. Diabetes 1989 38 1165–1174. (10.2337/diab.38.9.1165) 2670645

[29] Diamanti-Kandarakis E Kouli Ch Alexandraki K Spina G. Failure of mathematical indices to accurately assess insulin resistance in lean, overweight, or obese women with polycystic polycystic ovary syndrome. Journal of Clinical Endocrinology and Metabolism 2004 89 1273–1276. (10.1210/jc.2003-031205) 15001622

[30] Cassar S Misso ML Hopkins WG Shaw CS Teede HJ Stepto NK. Insulin resistance in polycystic ovary syndrome: a systematic review and meta-analysis of euglycaemic-hyperinsulinaemic clamp studies. Human Reproduction 2016 31 2619–2631. (10.1093/humrep/dew243) 27907900

[31] Lunger F Wildt L Seeber B. Accurate screening for insulin resistance in PCOS women using fasting insulin concentrations. Gynecological Endocrinology 2013 29 541–544. (10.3109/09513590.2013.774362) 23464983

[32] Muniyappa R Irving BA Unni US Briggs WM Nair KS Quon MJ Kurpad AV. Limited predictive ability of surrogate indices of insulin sensitivity/resistance in Asian-Indian men. American Journal of Physiology: Endocrinology and Metabolism 2010 299 E1106–E1112. (10.1152/ajpendo.00454.2010) 20943755 PMC3006259

[33] Kanauchi M Tsujimoto N Hashimoto T. Validation of simple indices to assess insulin sensitivity based on the oral glucose tolerance test in the Japanese population. Diabetes Research and Clinical Practice 2002 55 229–235. (10.1016/S0168-8227(01)00313-8) 11850099

[34] Hücking K Watanabe RM Stefanovski D Bergman RN. OGTT-derived measures of insulin sensitivity are confounded by factors other than insulin sensitivity itself. Obesity 2008 16 1938–1945. (10.1038/oby.2008.336). 18670420 PMC3417105

[35] Ruige JB Mertens IL Bartholomeeusen E Dirinck E Ferrannini E Van Gaal LF. Fasting-based estimates of insulin sensitivity in overweight and obesity: a critical appraisal. Obesity 2006 14 1250–1256. (10.1038/oby.2006.142). 16899806

[36] Chrenova J Rausova Z Penesova A Dedik L. Comparison of insulin sensitivity indices properties calculated from OGTT. Central European Journal of Medicine 2011 6 567–574. (10.2478/s11536-011-0056-7)

